# Improving Real-Time Hand Gesture Recognition with Semantic Segmentation

**DOI:** 10.3390/s21020356

**Published:** 2021-01-07

**Authors:** Gibran Benitez-Garcia, Lidia Prudente-Tixteco, Luis Carlos Castro-Madrid, Rocio Toscano-Medina, Jesus Olivares-Mercado, Gabriel Sanchez-Perez, Luis Javier Garcia Villalba

**Affiliations:** 1 Department of Informatics, The University of Electro-Communications, Chofu-shi 182-8585, Japan; gibranbg@uec.ac.jp; 2 Instituto Politecnico Nacional, ESIME Culhuacan, Mexico City 04440, Mexico; lprudente@ipn.mx (L.P.-T.); lcastro@ipn.mx (L.C.C.-M.); rtoscanom@ipn.mx (R.T.-M.); jolivares@ipn.mx (J.O.-M.); gsanchez@ipn.mx (G.S.-P.); 3 Group of Analysis, Security and Systems (GASS), Department of Software Engineering and Artificial Intelligence (DISIA), Faculty of Computer Science and Engineering, Universidad Complutense de Madrid (UCM), Calle Profesor José Garcia Santesmases, 28040 Madrid, Spain

**Keywords:** hand gesture recognition, hand segmentation, FASSD-Net, TSN, TSM

## Abstract

Hand gesture recognition (HGR) takes a central role in human–computer interaction, covering a wide range of applications in the automotive sector, consumer electronics, home automation, and others. In recent years, accurate and efficient deep learning models have been proposed for real-time applications. However, the most accurate approaches tend to employ multiple modalities derived from RGB input frames, such as optical flow. This practice limits real-time performance due to intense extra computational cost. In this paper, we avoid the optical flow computation by proposing a real-time hand gesture recognition method based on RGB frames combined with hand segmentation masks. We employ a light-weight semantic segmentation method (FASSD-Net) to boost the accuracy of two efficient HGR methods: Temporal Segment Networks (TSN) and Temporal Shift Modules (TSM). We demonstrate the efficiency of the proposal on our IPN Hand dataset, which includes thirteen different gestures focused on interaction with touchless screens. The experimental results show that our approach significantly overcomes the accuracy of the original TSN and TSM algorithms by keeping real-time performance.

## 1. Introduction

Hand gesture recognition (HGR) plays a central role in Human–Computer Interaction (HCI). Recently, HGR systems using vision-based interaction and control have become more common [1,2,3], and they are, compared to the conventional inputs of mouse and keyboard, more natural because of the intuitiveness of hand gestures. Therefore, HGR dominates a wide range of applications in the automotive sector, consumer electronics, home automation, and others [3,4,5,6]. An essential feature for these applications is real-time performance, so that HGR systems must be designed to give feedback with no lag to the gestures that users may input. In particular, touchless screen manipulation is an application that requires no lag in the HGR, so that users can be able to manipulate interfaces and control the location of the cursor in real-time [7,8,9]. Therefore, a compact and efficient HGR system is necessary to fulfill the requirements of real-time applications.

In recent years, accurate and efficient deep learning models have been proposed for HGR [9,10,11,12,13]. Multiple modality inputs are used to further improve the performance of these CNN-based models. Depth [10,14] and optical flow [11,15] are the most common complements to the RGB images. However, the depth modality needs an extra sensor to capture accurate information from the user’s hands. On the other hand, dense optical flow is an expensive computational process that limits real-time performance. Nonetheless, there are recent approaches that achieve fast inference speed.

**Figure 1:**
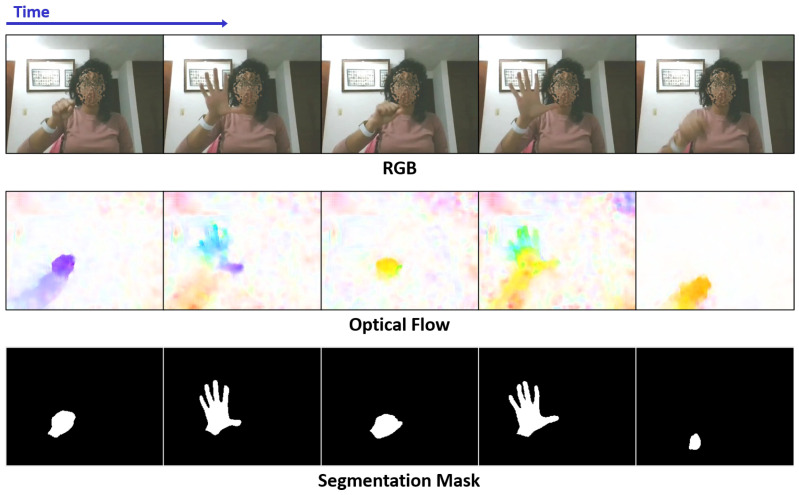
Example of the three different modalities analyzed in this paper. Note that segmentation masks and optical flow images can be performed in real-time using FASSD-Net [16] and SpyNet [19], respectively.

In this paper, we propose to use hand segmentation masks as an alternative to depth and optical flow modalities. Specifically, we extend our FASSD-Net [16] algorithm of real-time semantic segmentation to classify hands pixel-wise accurately. Besides, we compare the contribution of semantic segmentation and optical flow on two efficient state-of-the-art (SOTA) approaches: Temporal Segment Networks (TSN) [17] and Temporal Shift Modules (TSM) [18]. Figure 1 shows an example of the three input modalities analyzed in this work. Note that dense optical flow is computed in real-time with 320×240 size images using the efficient Spatial Pyramid Network (SpyNet) [19].

To validate our proposal, we composed the IPN Hand dataset [20] for hand gesture recognition. The dataset contains about 800 thousand frames from more than four thousand RGB gesture videos performed by 50 distinct subjects. Our dataset includes thirteen classes of two gesture types (static and dynamic) designed for interaction with touchless screens. Some of the challenges that this dataset introduces are different scenes, including clutter backgrounds, strong and weak illumination conditions, as well as static and dynamic background environments. Besides, to accurately train semantic segmentation models, we manually annotate (pixel-level) the hands’ location and the user shape of 500 frames of the dataset. With the IPN Hand dataset, we experimentally prove that the combined modality of RGB-S (segmentation) is comparable and even better than that of RGB-OF (optical flow) for HGR based on both TSN and TSM approaches. Furthermore, the RGB-S approach is significantly faster than its OF pair, keeping the real-time requirement of HGR applications.

In summary, the main contributions of this paper are:

- We propose an alternative to the expensive dense optical flow estimation, and the extra sensor requirement of depth images, by using semantic segmentation images of the hand for real-time hand gesture recognition.
- By using a simple input modality combination, we demonstrate that our RGB-S proposal outperforms state-of-the-art methods of TSN and TSM. We also present a statistical analysis of both approaches’ results, which shows the type of gestures that gets more benefit from different input modalities.
- We extend our new IPN Hand dataset [20], including pixel-level annotations useful for training semantic segmentation models. Currently available at github.com/GibranBenitez/IPN-hand.

## 2. Related Work

HGR methods can be divided into two groups based on the way for obtaining the gesture features, i.e., e hand-crafted and deep-network based approaches [7,8,21]. Hand-crafted features are usually extracted using descriptors such as histogram of oriented gradients (HOG) and histogram of optical flow (HoOF) [21,22,23,24]. For instance, Ohn-Bar et al. [22] evaluated different variations of HOG and linear classifiers for HGR applied to automotive interfaces. Joshi et al. [23] used HOG and Random Forest (RF) for classifying upper body gestures. Borghi et al. [24] employed HoOF and support vector machines (SVM) for modeling the gestures in a spatio-temporal feature space. Note that HoOF features are based on the computational expensive dense optical flow (OF) estimation, which extracts temporal information from adjacent frames. On the other hand, Contreras and Gallegos [25] avoid HoOF estimation and propose a hand segmentation technique based on HSV and CIELab color space for single dynamic gesture recognition. This approach can run in real-time using PCA and KNN for feature extraction and classification, respectively. However, it is limited to dynamic gestures with low temporal information.

On the other hand, previous works focused on learned feature extraction methods tend to use deep convolutional neural networks (CNNs) [6,12,26] and 3D-CNNs [10,11,13,27] with a variety of different input modalities, such as RGB, depth [6], OF [15], infrared [26], and even surface electromyography signals [28,29] (using armbands to sense electrical activity of skeletal muscles). Specifically, multi-stream architectures based on different versions of the same video with two or more CNNs in parallel, have been widely employed [9,10,11,12,13,26,27]. The seminal work of Karpathy et al. [30] establishes the trend of using two-stream architectures for action recognition, which originally combines features from low-resolution frames and high-resolution cues from the center of the frame. Concurrently, the pioneering work of Simonyan et al. [31] first introduces the multimodal fusion of two different features, with one stream dedicated to RGB images and the other with OF fields.

The multi-stream technique has been prevalent in recent years as shown in the ChaLearn Look At People (LAP) gesture recognition challenge [32], where all the entries used multi-stream architectures of at least RGB and depth streams. The winners of the last IsoGD challenge, Miao et al. [10], employ a well-known 3D-CNN model called C3D [33] to extract features from RGB, depth, and OF fields. They propose a feature level fusion within each modality, and use SVM for classifying the fused features. One year after that challenge, Narayana et al. [12] overcome the results by proposing a late-fusion approach from 12 different channels, comprising focus regions of global, left and right hand, including modalities of RGB, depth, and OF. Recently, Hakim et al. [27] proposed to fuse RGB and depth spatio-temporal features (extracted with 3D-CNNs and LSTM recurrent neural networks) with a Finite State Machine that restricts some gesture flows and limits the recognition classes. D’Eusanio et al. [26], on the other hand, proposed an early-fusion approach of RGB, depth, and infrared modalities based on a modification of the very deep DenseNet-161 architecture. Concurrently, Kopuklu et al. [13] proposed a hierarchical structure of 3D-CNN architectures to detect and classify continuous hand gestures. A shallow 3D-CNN model discriminates between gestures and non-gestures in the detection stage, while the recognition is carried out by a deep 3D-CNN using weighted average filtering to take a single-time activation per gesture. In contrast to those methods, we focus on real-time performance. Therefore, we used a 2D-CNN as compact as possible. Besides, based on an efficient semantic segmentation process, we present an alternative to the computational expensive OF estimation, as well as the use of extra sensors for depth maps.

## 3. Proposed Method

In this section, we introduce the FASSD-Net [16] algorithm for real-time semantic segmentation used to classify hands pixel-wise, as well as, the two efficient HGR approaches which are based on our proposed multimodal RGB-S input, Temporal Segment Networks (TSN) [17], and Temporal Shift Modules (TSM) [18].

### 3.1. FASSD-Net for Real-Time Semantic Segmentation

**Figure 2:**
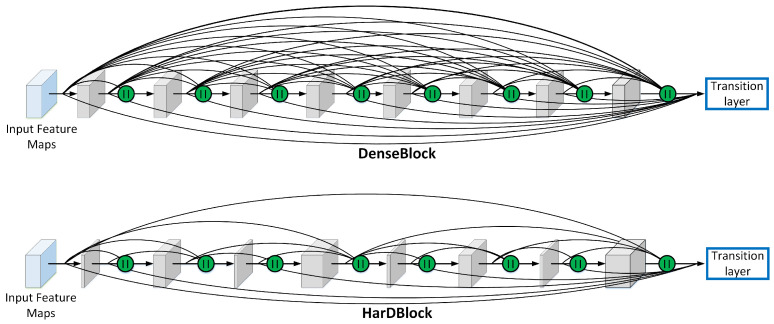
Concatenation scheme comparison between the DenseBlock and the HarDBlock. The HarDBlock is named after the harmonic wave patterns that describe its concatenation scheme. “||” denotes concatenation process.

FASSD-Net [16] is based on the Harmonic Dense-Net architecture (HarDNet) [34], a recent SOTA network inspired by Densely Connected Network (DenseNet) [35]. Its core component, the HarDBlock (Harmonic Dense Block), is specifically designed to address the memory traffic problems of the DenseBlock, and it is optimized to increase the density of computations of the layers, as shown in Figure 2. We increase the segmentation performance by introducing two key modules to the U-shape encoder-decoder version of HarDNet. The first module, Dilated Asymmetric Pyramidal Fusion (DAPF), is designed to increase the receptive field on the top of the last stage of the encoder. The second module, Multi-resolution Dilated Asymmetric (MDA) module, fuses and refines detail and contextual information from multi-scale feature maps coming from early and deeper stages of the network. Both modules are designed to keep a low computational complexity by using asymmetric convolutions. The FASSD-Net is capable of running at 38 fps with an input size of 512 × 1024 pixels on the low-power consumption Jetson Xavier NX SBC. Table 1 shows the FASSD-Net architecture used to segment pixels into four classes: background, human shape, left and right hands. The DAPF module is located right after the last encoder block (B4), while MDA modules are used to fuse feature maps from different scales before each decoder block.

### 3.2. Temporal Segment Networks (TSN)

The pioneering work of Wang et al. [17] proposes to perform video-level predictions from entire videos using the visual information extracted from 2D-CNNs only. Instead of applying the 2D-CNN model on single frames, the proposed Temporal Segment Networks (TSN) operate on a sequence of short clips sparsely sampled from the entire video. Individual preliminary predictions are produced by each short clip in the sequence based on the defined gesture classes. Then the final video-level prediction is defined as a consensus among all short clips. One of TSN’s key contributions resides in the learning process, which is based on the loss values of video-level predictions instead of those from the short clips. Thus, the model parameters are iteratively updated based on the error produced at the video-level.

**Figure 3:**
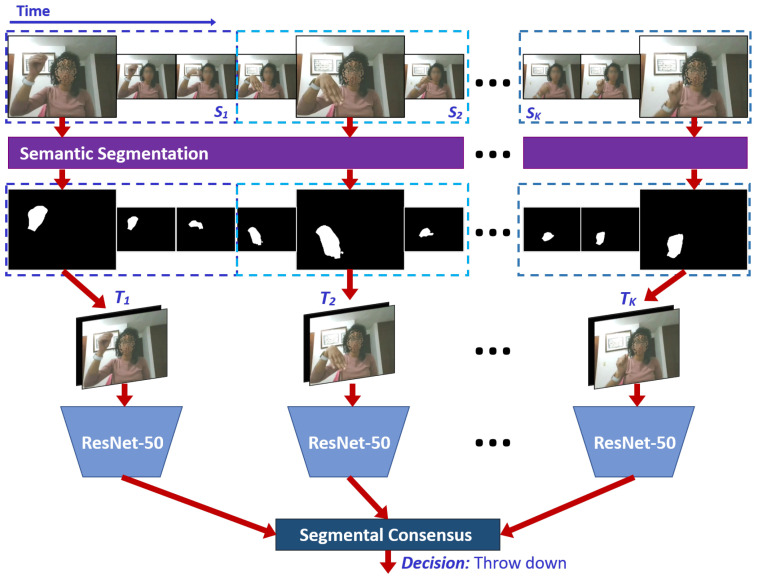
Temporal Segment Networks (TSN) process.

The original proposal of TSN presents a two-stream CNN combining the consensus of RGB-based and OF-based networks. In our proposal, instead, we propose to exclude the two-stream approach by using a single CNN based on an RGB-S input, as shown in Figure 3. In this paper, we used ResNet-50 [36] as the main 2D-CNN architecture of TSN. Formally, given a video *V*, we divide it into *K* segments ($S_{k}$) of an equal number of frames. Then, the FASSD-Net is used to obtain segmentation masks of each frame to define the multimodal RGB-S input. Finally, the TSN applied to a sequence of short clips is defined as follows:

(1) $$TSN\left(T_{1},T_{2},...,T_{K}\right)=H\left(G\left(F\left(T_{1};W\right),F\left(T_{2};W\right),...,F\left(T_{K};W\right)\right)\right),$$

where ($T_{1},T_{2},...,T_{K}$) is a sequence of short clips. Each $T_{k}$ is randomly sampled from its corresponding segment $S_{k}$. *F* represents the CNN with parameters W and produces class scores for all the classes. The consensus function *G* averages the class scores from multiple short clips. Based on this consensus, the Softmax function *H* predicts the probability of each action class for the whole video. Note that there is a single CNN model with shared parameters W among multiple short clips, which are jointly optimized based on the video-level predictions with standard back-propagation algorithms.

### 3.3. Temporal Shift Module (TSM)

**Figure 4:**
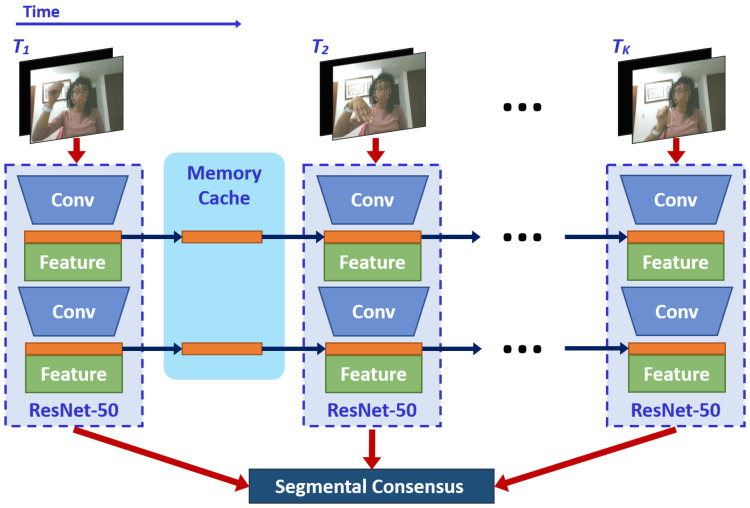
TSM process.

The main drawback of the 2D-CNN-based networks is that extracted low-level features cannot capture the temporal information needed for HGR. Since the model is learned frame-wise in each short clip, it cannot infer the temporal order or more complicated temporal relationships. Recently, Lin et al. [18] introduced the temporal shift module (TSM) as a simple yet effective solution to this problem. In particular, it shifts part of the channels along the temporal dimension, facilitating information exchanged among neighboring frames. As shown in Figure 4, TSM can be inserted into 2D-CNNs to achieve temporal modeling similar to 3D-CNNs, at negligible computational cost without learning extra parameters.

Following its original implementation [18], we insert TSM on the TSN framework, enabling temporal information fusion at no computation. So that, a 3D-CNN process is emulated by inserting a temporal shift module on each convolution block, enlarging the temporal receptive field as if running a convolution along the temporal dimension. The TSM-based network learns the parameters using a sequence of short clips as described in Equation (1). Furthermore, to fulfill the online requirements, for each frame, the first 1/8 feature maps of each residual block of ResNet-50 [36], are stored in the memory cache. Subsequently, the first 1/8 feature maps of the next frame are replaced with those previously stored. In resume, the combination of 7/8 current, and 1/8 old feature maps are used to generate each convolutional layer. Figure 4 shows the diagram of our TSM-based network, where we further increase the feature extraction capability by providing the hand segmentation masks at the data-level.

## 4. Experimental Results

In this section, we present the dataset we used for evaluating our proposal, as well as the implementation details, and the experimental results.

### 4.1. Dataset

**Figure 5:**
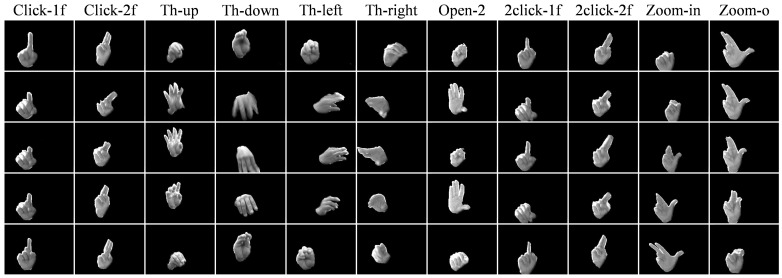
Examples of the 11 action gestures included in the IPN Hand dataset. The temporal order is shown from top to down rows. For visualization purposes, segmented hand masks were blended to the RGB images. Note that not shown here are the pointing and the non-gesture samples.

**Figure 6:**
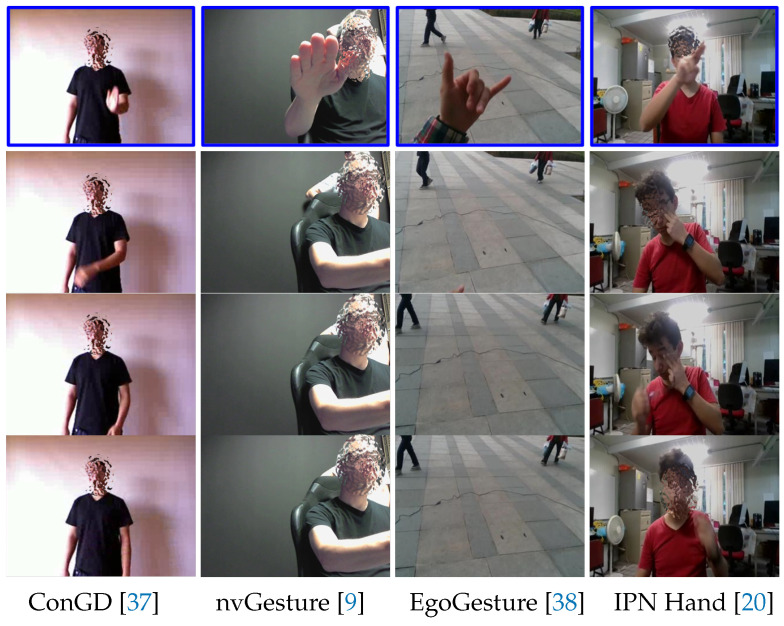
Comparison of gesture vs. non-gesture frames between commonly used hand gesture datasets and the IPN Hand. The first row (blue) shows gesture frames, while the second to fourth rows show non-gesture frames.

In this paper, we used the new IPN Hand dataset [20], which includes more than 5000 instances of static and dynamic gestures designed for the interaction with touchless screens. In particular, it includes gestures to control the pointer’s location on the screen (pointing), and to manipulate interfaces (actions): for the former, two static gestures of pointing with one and two fingers are included (Point-1f & Point-2f); for the latter, 11 gestures are defined, including click with one and two fingers (Click-1f & Click-2f), throw to four positions (Th-up, Th-down, Th-left & Th-right), open the hand twice (Open-2), double click with one and two fingers (2click-1f & 2click-2f), zoom-in, and zoom-out (Zoom-in & Zoom-o), as shown in Figure 5. Segments where natural hand movements are performed between target classes, are defined as non-gestures states (No-gest). Non-gesture segments represent the largest number of instances in the dataset (1431). On the other hand, pointing classes include around 1000 instances each, while action classes 200 per gesture. It is worth noting that, the IPN Hand dataset presents a more realistic and challenging situation compared to common datasets due to the nature of non-gesture states, which show natural hand movements similar to some of the target gestures. Figure 6 compares gesture vs. non-gesture states between the IPN Hand and commonly used hand gesture datasets, such as ConGD [37], nvGesture [9], and EgoGesture [38]. As we can see, the non-gesture states of the IPN Hand dataset are significantly more challenging to exclude compared to other datasets, which makes an excellent evaluation point for HGR models.

**Figure 7:**
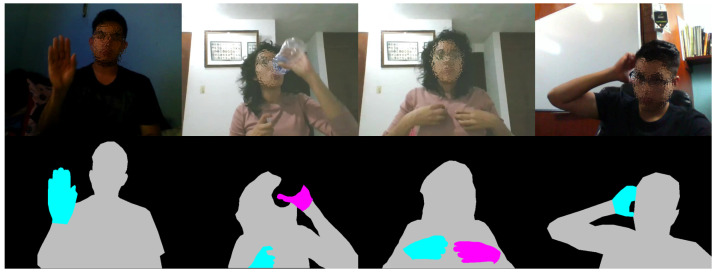
Example of the pixel-level annotations on non-gesture frames. Top row: original RGB frames. Bottom row: pixel-level annotations, gray=person, cyan=right, magenta=left hand.

The dataset comprises 200 long videos performed by 50 different subjects. The start and end frame index of each gesture instance in these videos were manually labeled. All instances were recorded with an RGB camera at a resolution of 640×480 with a frame rate of 30 fps. The average duration of action gestures is 65 frames, with a minimum of 9, and a maximum of 112 frames. On the other hand, the average duration of pointer classes and non-gesture clips is 220 and 150 frames, respectively. In addition to the class-label annotations, for this paper, we manually annotate 500 frames at pixel-level. In this way, semantic segmentation algorithms can be trained to detect hands and humans, in realistic scenarios. Specifically, we define the segmentation labels of person, left, and right hands, as shown in Figure 7. The complete dataset with our annotations is now available at github.com/GibranBenitez/IPN-hand.

We use the originally proposed data split [20], which is divided by subject into training (74%), and testing (26%) sets, resulting in 148 and 52 videos, respectively. The amount of pointer/action gesture instances in training, and testing splits are 3117, and 1101, respectively. In this way, we ensure a strict evaluation since none of the subjects included in the testing sets are shown in training.

### 4.2. Implementation Details

We use Python 3.6 and PyTorch 1.2 with CUDA 10.2 for all experiments, and the inference time of OF and segment calculation is measured on an Intel Core i7-9700K desktop with a single Nvidia GTX 1080Ti GPU, if not specified otherwise.

We train the FASSD-Net [16] model with a synthetic hand pose estimation dataset [39], which contains more than 40 thousand images with hands fully annotated at pixel-level (https://lmb.informatik.uni-freiburg.de/projects/hand3d/). Following the original open-source implementation of FASSD-Net [16], we use cross-entropy loss, as well as Stochastic Gradient Descent (SGD) with weight-decay 5e-4 and momentum 0.9. The “poly” learning rate strategy is used with an initial learning rate of 0.02. We train the model on a multi-GPU setup with two Nvidia GTX 1080Ti. Thus, based on the GPU memory limitations, the training was set for 90k iterations with batch size 32 and a crop size of 480×360. With this model, we fine-tune the part of the IPN Hand dataset that we annotated pixel-wise, where 400 and 100 frames were randomly selected for training and validation, respectively. We extend the same training protocol for 45000 iterations for the final model, setting the batch size to 64 and the learning rate to 0.001. Finally, we obtain the complete dataset’s segmentation masks at the original input size of 640×480, where hand masks are binarized, as shown in Figure 1. On the other hand, the dense optical flow estimation is obtained with the SPyNet. We used the open-source implementation and pre-trained model of [40] to calculate real-time OF maps for the complete dataset. Following common standards, we employ the color wheel described in [41] for color-coding the optical flow values, as shown in Figure 1. To keep the RGB-OF input size consistent, we convert the color-coded OF to grayscale images. Note that, for reaching real-time performance, we resized to 320×240 the input for SPyNet, while we use the original input size of 640×480 for the FASSD-Net.

We used the same ResNet-50 [36] backbone on both TSN, and TSM, which is pre-trained on the ImageNet dataset [42]. The input size of the IPN Hand video frames is resized to 320×240 to achieve faster computation. Concerning data augmentation, we use random scaling of (1×,0.875×,0.75×,0.66×) and random cropping. Following the original open-source implementation of TSM [18], the training parameters are based on the number of segments (*K*). Specifically, for K=32 are: 60 training epochs, initial learning rate of 1e-4 (decays by 0.1 at epochs 20 & 40), weight decay 5e-4, batch size 24, and dropout 0.5. We defined the batch size based on the memory restrictions of the same setup of the semantic segmentation model. Furthermore, for the lower number of segments, we modify the batch size and the learning rate accordingly.

### 4.3. Evaluation Metrics

All the dataset videos are segmented into isolated gesture samples based on the beginning and ending frames manually annotated. So that, the classification problem resided on predicting the class label for each gesture sample. In this paper, the percent of correctly labeled samples (Accuracy), and the confusion matrix of all isolated gesture predictions are used as evaluation metrics. Nonetheless, the number of segments and the number of frames of each gesture sample restricts the one-to-one evaluation. Therefore, a common practice is to sample 10 random multiple clips per video [18,43]. However, this protocol cannot fairly evaluate a big difference in the length of different clips nor gestures that are repetitive, such as double clicks or open twice classes. Therefore, we propose to use a controlled sliding window instead of random multiple clips evaluation. So that, the predicted label of a video *V* with duration *N* is based on the average prediction scores of 10 clips of *K* frames, sparsed by a stride of size *S*, where S=max(1,(N/K)/K). We use this protocol to calculate classification accuracy and confusion matrices.

### 4.4. Results with Different Number of Segments

Table 2 shows the comparison of the results of TSM using a different number of segments (*K*) with RGB and RGB-S modalities. From this table, we can see that the proposed RGB-S outperforms RGB’s accuracy for all the choosing segments. The improvement is more significant when choosing 32 segments for the process. In this way, RGB-S with 32 frames achieves the best accuracy when using TSM.

**Figure 8:**
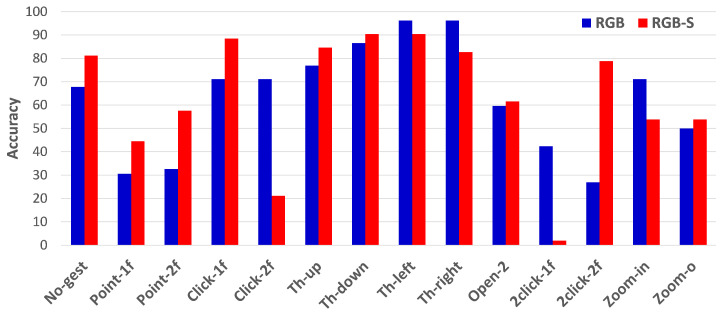
Per-class accuracy performance of TSM with 32 segments using RGB and RGB-S modalities.

To clearly show the improvement achieved of RGB-S, Figure 8 presents per-class accuracy with both input modalities. Not all the classes are improved at the same rate, and some are even better when using only RGB. The most significant improvement is achieved for the pointing classes and the non-gesture, while classes of throw right and zoom-in are clearly better when using the RGB modality. Interestingly, the results of clicking gestures present contrasting results for the two modalities. Click with one finger, and double click with two fingers are easily classified when using RGB-S, while RGB achieves the opposite situation. Nonetheless, the results of RGB are more stable in these classes. In general, the average accuracy per class for each modality is 62.8%, 63.6% for RGB and RGB-S, respectively. Note that these results are different from the accuracy performance shown in Table 2 due to the samples’ imbalance for each class. As mentioned before, non-gesture and pointing classes have considerably more samples than the action classes. Therefore, we separate the evaluation results of the IPN Hand dataset into two setups: when classifying the complete dataset (13 classes plus non-gesture clips); and when using the 11 action classes only.

### 4.5. Results Using the Complete Dataset

In this sub-section, we compare the results of TSM and TSN when using different input modalities with 32 segments. Table 3 shows the performance of both methods using 32 segments. RGB-OF is also compared in this table. To show the general class performance, the average accuracy per class is also included. This table shows that the RGB-S modality outperforms RGB and is comparable to RGB-OF specifically for the average class accuracy. In this way, we prove that RGB-S is a suitable alternative to improve RGB modality. Furthermore, the inference time of the frame-wise calculation for semantic segmentation is considerable faster than that for OF. Particularly, semantic segmentation with FASSD-Net takes 8 ms, while SpyNet needs 29 ms to calculate dense optical flow. Rather than the model (TSM vs. TSN), the extra modality latency directly impacts the total inference time for multi-modal models, being RGB-seg significantly faster (approx. 66 fps) than RGB-flo (approx. 28 fps). Note that, as discussed in the implementation details, FASSD-Net uses the original input size of 640×480 pixels, while for SpyNet, the input must be resized to 320×240 for reaching real-time performance.

**Figure 9:**
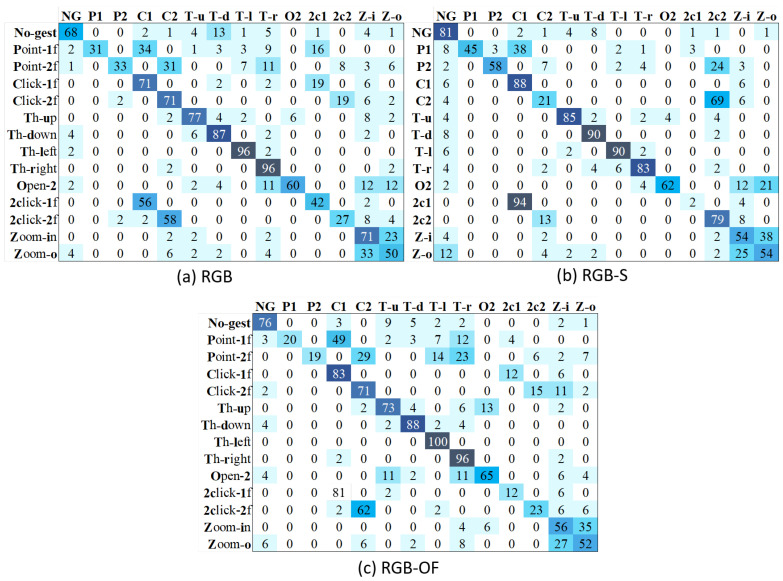
Confusion matrices of TSM with different input modalities.

**Figure 10:**
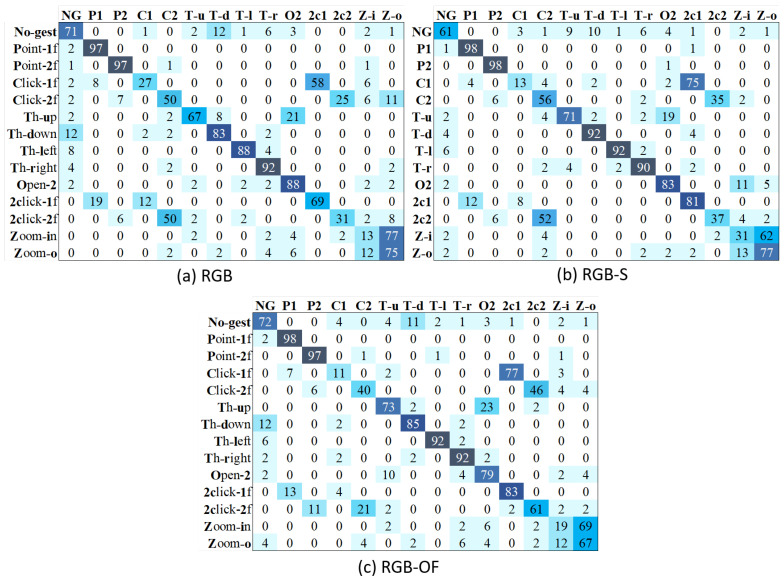
Confusion matrices of TSN with different input modalities.

Interestingly, the accuracy of TSN is significantly better than that of TSM. To clearly analyze each method’s performance, we present its confusion matrices with the different modalities. Figure 9 shows the results of TSM, where we can see that RGB’s major problems are related to the misclassification of non-gestures and pointing with several classes. RGB-OF partially solves these, while RGB-S clearly improves the accuracy. However, the misclassification problems between zoom-in and zoom-out classes are less severe on the RGB modality. On the other hand, Figure 10 illustrates the confusion matrices of different modalities for TSN. Contrary to TSM, this model does not present a problem to recognize the pointing classes, solution attributed to the method itself since all modalities show similar results. Besides, RGB-S achieves the best results for throwing classes. Resulting in higher average performance than other modalities.

Comparing Figure 9 and Figure 10, we can see a clear difference in the accuracy of pointing classes. However, there is no clear evidence of superiority in the action classes. This situation is further analyzed in the next sub-section.

### 4.6. Results Using Action Classes of the Dataset

In this experiment, we train the models analyzed in the previous sub-section with only the 11 action classes of the dataset. In this way, we can evaluate the performance of both HGR methods, as well as the improvement gained by different multimodality. Table 4 shows the performance of both methods with the three modalities. Note that the average accuracy per class is the same as the general accuracy performance due to the 11 action classes have the same number of samples. Contrary to the previous test, no imbalance is presented. From this table, we can see that TSN still achieves the best performance. However, opposite to TSN, it is evident that TSM achieves considerably better accuracy than the test shown in Table 3. These results indicate that TSN is better to classify static gestures than TSM. Furthermore, the improvement of our RGB-S proposal is more evident in this test, where TSN with RGB-S achieves 68%, the best accuracy.

**Figure 11:**
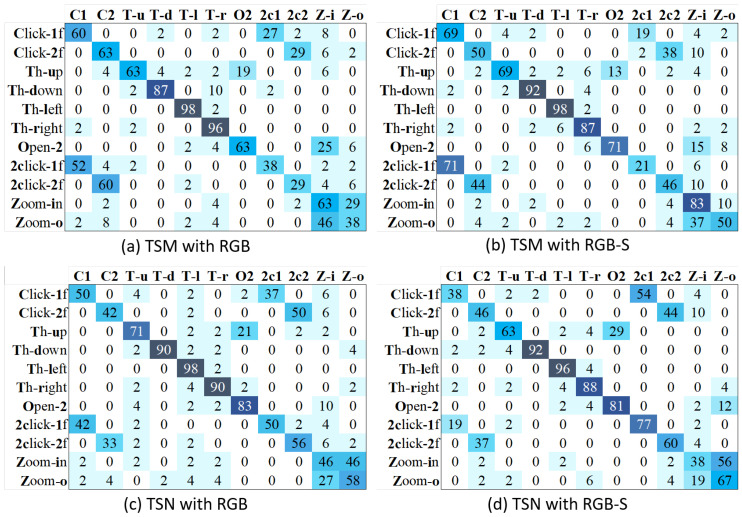
Confusion matrices of TSM and TSN with different input modalities, using action classes only.

Figure 11 shows the confusion matrices of TSM and TSN methods with RGB and RGB-S modalities. We can see that our proposed RGB-S helps to solve problems related to a similar appearance in hand shapes such as Open-2→Zoom-in for TSM, and Zoom-o→Zoom-in for TSN. However, both methods present evident problems to distinguish between clicking and double-clicking classes, a problem not solved even with multimodal inputs. We speculate the reason is that repetitive gestures such as open twice and double-clicking, may need a longer time span to be evaluated correctly. In order words, 32 frames may not be enough to cover the main activity of these classes.

## 5. Discussion

In the previous section, we have shown that our proposed RGB-S modality helps to improve the accuracy performance of both TSM and TSN methods. However, we obtain interesting results when comparing both methods using the same modality. TSM [18] was proposed as a direct improvement to the well-known TSN [17] method. Nonetheless, our experimental results show that TSM performs worse than TSN for hand gesture recognition using our IPN Hand dataset. Thus, in this section, we discuss the possible reasons for this situation.

First of all, as mentioned in Section 4.5, the number of samples per class is imbalanced. The test set comprises 1610 segmented videos that include one gesture each. Specifically, in the test set, we have 508 clips for the non-gesture class and 265 clips for each pointing class (Point-1f & Point-2f), while there are only 52 clips per action class. Therefore, the test set average accuracy will be higher for the method that recognizes better the pointing classes and non-gestures. That is exactly the case of TSN. In this way, we speculate that TSN results are high for these classes because the model relies more on appearance than on temporal information. In other words, if a person is moving his hand while pointing with one finger, that motion (temporal information) might be misclassified with the action of clicking with one finger. Since TSM includes temporal information of all convolutional blocks, it has more chance to fail on recognizing those classes.

**Figure 12:**
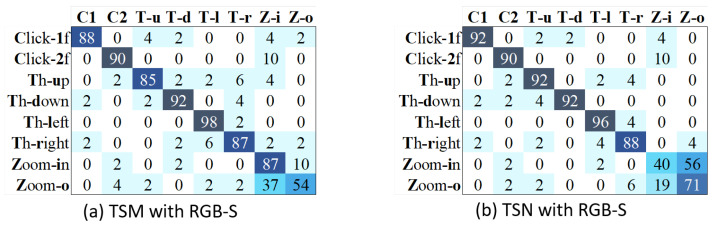
Confusion matrices of TSM and TSN are estimated by excluding the repetitive classes of open twice and double-clicking.

On the other hand, the problem of repetitive gestures mentioned in Section 4.6 may worsen the performance of TSM. To prove that, we estimate the results of a short action class set by excluding the repetitive gestures (Open-2, 2click-1f & 2click-2f). Figure 12 shows the confusion matrices of both methods using our RGB-S proposal. As expected, the results of TSM are higher than those of TSN, with an average accuracy of 85.1% and 82.9% for TSM and TSN, respectively. In this figure, we can see that the problems of TSM for recognizing clicking gestures are solved. Besides, the TSM can handle better than TSN the similar appearance problems related to zoom-in and zoom-out gestures. In this way, we can conclude that TSM performs better for gestures that rely more on temporal information, while TSN achieves good for static hand gestures.

## 6. Conclusions

In this paper, we proposed an alternative to the expensive dense optical flow estimation used in the multimodal approaches for hand gesture recognition (HGR). We based our proposal on a combination of RGB frames with hand segmentation masks. To this end, we employed a light-weight semantic segmentation method (FASSD-Net) to boost the accuracy of two real-time HGR methods: Temporal Segment Networks (TSN) and Temporal Shift Modules (TSM). We demonstrated the efficiency of RGB-S modality over RGB-OF and RGB with extensive experimental tests. Furthermore, we analyze both HGR methods’ results, showing that TSM is better at recognizing gestures that rely on temporal information, while TSN stands out on static gestures. As future work, we plan to combine TSM and TSN in a single architecture to recognize both types of hand gestures in real-time.

## Figures and Tables

**Table 1:** Example of the FASSD-Net architecture with a 512 × 1024 input.

Stage	Name	Type	Output Size
Input	-	-	512 × 1024 × 3
Encoder	Stem Conv	Conv 3 × 3 (s=2)	256 × 512 × 16
		Conv 3 × 3	256 × 512 × 24
		Conv 3 × 3 (s=2)	128 × 256 × 32
		Conv 3 × 3	128 × 256 × 48
	Encoder B1	HarDBlock (L=4)	128 × 256 × 64
	Encoder B2	2D Average Pooling	64 × 128 × 96
		HarDBlock (L=4)	
	Encoder B3	2D Average Pooling	32 × 64 × 160
		HarDBlock (L=8)	
	Encoder B4	2D Average Pooling	16 × 32 × 224
		HarDBlock (L=8)	
	DAPF	-	16 × 32 × 224
Decoder	MDA	-	32 × 64 × 192
	Decoder B1	HarDBlock (L=8)	32 × 64 × 160
	MDA	-	64 × 128 × 119
	Decoder B2	HarDBlock (L=4)	64 × 128 × 78
	MDA	-	128 × 256 × 63
	Decoder B3	HarDBlock (L=4)	128 × 256 × 48
	Output Conv	Conv 1 × 1	128 × 256 × 4
		Upsampling × 4	512 × 1024 × 4

*L* denotes the number of convolution layers in the HarDBlock, and *s* represents the stride of the convolution.

**Table 2:** Temporal Shift Module (TSM) results with different number of segments using RGB and RGB-S modalities.

Modality	Segments	Accuracy
RGB	8	54.0373
RGB	16	50.4969
RGB	24	55.0932
RGB	**32**	**55.9627**
RGB-S	8	55.5901
RGB-S	16	50.559
RGB-S	24	55.3478
RGB-S	**32**	**65.2795**

Bold and underline denotes the best result, while bold denotes the runner-up.

**Table 3:** Results of TSM and TSN with different modalities using the complete IPN Hand dataset.

Model	Segments	Modality	Extra Modality Time	Total Inference Time	Accuracy	Avg. Class Accuracy
TSM	32	RGB	-	6.8 ms (147 fps)	55.9627	62.7856
TSM	32	**RGB-seg**	8 ms	15.2 ms (66 fps)	**65.2795**	**63.6383**
TSM	32	RGB-flo	29 ms	36.2 ms (27 fps)	53.6646	59.5857
TSN	32	RGB	-	6.6 ms (152 fps)	76.2733	67.7925
TSN	32	**RGB-seg**	8 ms	15.1 ms (66 fps)	74.8447	**70.0014**
TSN	32	**RGB-flo**	29 ms	36.1 ms (28 fps)	**77.2671**	69.1529

Bold and underline denotes the best result, while bold denotes the runner-up.

**Table 4:** Results of TSM and TSN with different modalities using the 11 action classes of the IPN Hand dataset.

Model	Segments	Modality	Accuracy	Avg. Class Accuracy
TSM	32	RGB	63.6364	63.6364
TSM	32	**RGB-seg**	**66.958**	**66.958**
TSM	32	RGB-flo	65.035	65.035
TSN	32	RGB	66.7832	66.7832
TSN	32	**RGB-seg**	**68.007**	**68.007**
TSN	32	RGB-flo	65.9091	65.9091

Bold and underline denotes the best result, while bold denotes the runner-up.

## Data Availability

Publicly available datasets were analyzed in this study. This data can be found here: github.com/GibranBenitez/IPN-hand.
